# Mucinous cystic adenoma with inflammatory cell infiltration around the splenic artery mimicking pancreatic cancer: a case report

**DOI:** 10.1007/s12328-020-01228-y

**Published:** 2020-09-09

**Authors:** Hiroaki Okuse, Reiko Yamada, Kyosuke Tanaka, Noriyuki Horiki, Yoshiyuki Takei

**Affiliations:** 1 Department of Gastroenterology, Suzuka Kaisei Hospital, Suzuka, Mie Japan; 2 Department of Gastroenterology and Hepatology, Mie University Hospital, 2-174 Edobashi, Tsu, Mie 514-8507 Japan; 3 Department of Endoscopy, Mie University Hospital, Tsu, Mie Japan

**Keywords:** Mucinous cystic neoplasm, Ovarian-like stroma, Inflammatory cell infiltration

## Abstract

A 45-year-old woman presented with upper abdominal and back pain. A cystic lesion in the pancreas and inflammation around the splenic artery were detected by computed tomography. Although imaging studies were difficult to exclude malignancy, pathological and cytological findings of a fine-needle aspiration showed no signs of malignancy. The patient was, therefore, followed-up for 3 months, during which time the cyst increased in size and developed a cyst-in-cyst structure. She was diagnosed with mucinous cyst neoplasm and underwent distal pancreatectomy. Histologically, the patient was diagnosed as low-grade mucinous cystic adenoma. Soft tissue shadows around the splenic artery were considered to indicate fibrosis and infiltration of inflammatory cells. After distal pancreatectomy, the patient has been uneventful with symptom resolution. This case highlights the potentially atypical presentation of mucinous cystic neoplasms with inflammatory cell infiltration around the splenic artery.

## Introduction

Mucinous cystic neoplasms (MCNs) are cystic tumors covered with mucus-producing epithelium, with a characteristic ovarian-like stroma. MCNs are relatively rare tumors, accounting for only 2–5% of exocrine pancreatic tumors and occurring most frequently in the distal pancreas in younger women [1]. MCNs often appear as solitary or multilocular elliptical or spherical cystic tumors, typically protruding from the pancreas, in the form of a so-called cyst-in-cyst, with a common thick fibrous cap covering multiple cysts [2].

Most MCNs are slow growing and asymptomatic [3]. Naveed et al. reported a typical clinical appearance characterized by epigastric heaviness and fullness (60–90%) or by an abdominal mass (30–60%), sometimes with nausea, vomiting (20–30%), and back pain (7–40%) [3]. Yamao et al. also reported that 51.4% of MCNs were asymptomatic, and 6.5% presented with acute pancreatitis [4].

We report a case of mucinous cystic adenoma (MCA) with inflammatory cell infiltration around the splenic artery which was confirmed by pathology. The present case showed atypical imaging findings at the first examination, including infiltration around the splenic artery located at the back of the unilocular cystic lesion. However, during a 3-month observation period, the lesion showed a cyst-in-cyst appearance, thus supporting a preoperative diagnosis of MCN. Although it was difficult to differentiate this case from malignancy at the first examination, the tumor demonstrated morphological changes throughout 3 months of observation.

## Case report

A 45-year-old woman with no previous illnesses presented to the referring hospital with continuous and intractable upper abdominal and back pain, which was unresolved by pharmacotherapy. Plain computed tomography (CT) showed a cystic lesion in the pancreatic body, and the patient was referred to our institution for further examination.

**Fig. 1:**
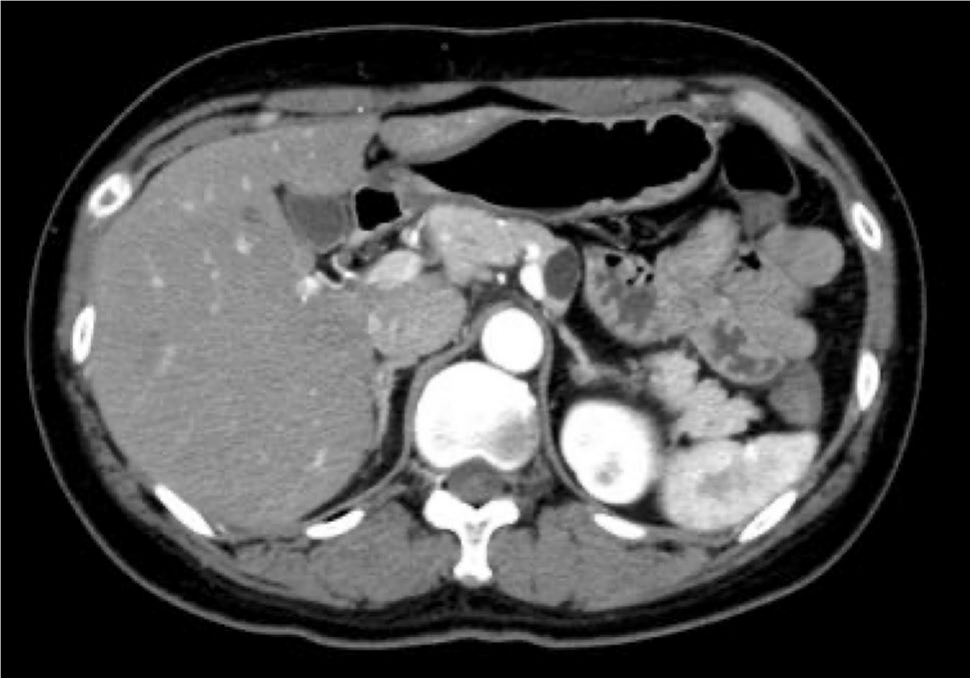
Unilocular cystic lesion in the pancreatic body detected 6 years before the current presentation

The cystic lesion in the pancreatic body had been noted 6 years previously at another hospital (Fig. 1). However, the patient was followed-up because the cyst was unilocular and small, and she had no symptoms at that time. However, 1 year previously, the patient revealed a history of epigastric pain occurring several times a month, but which had resolved spontaneously. She smoked 17 cigarettes a day, but did not drink. She had no family history of pancreatic diseases. Physical examination revealed moderate epigastric tenderness. Laboratory parameters, including amylase, lipase, and C-reactive protein, and tumor markers, including carcinoembryonic antigen (CEA), carbohydrate antigen (CA19-9), pancreatic cancer-associated antigen-2, and elastase-1, were all within normal ranges.

**Fig. 2:**
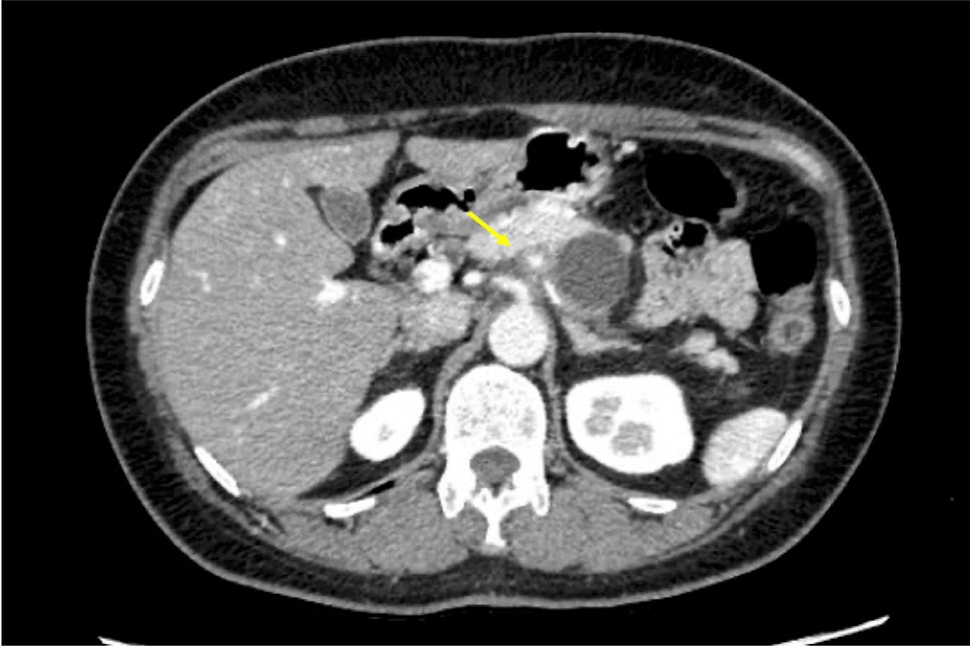
Enhanced computed tomography revealed a large 3-cm unilocular cystic lesion in the pancreatic body and a soft tissue lesion around the splenic artery on the papillary side (yellow arrow)

**Fig. 3:**
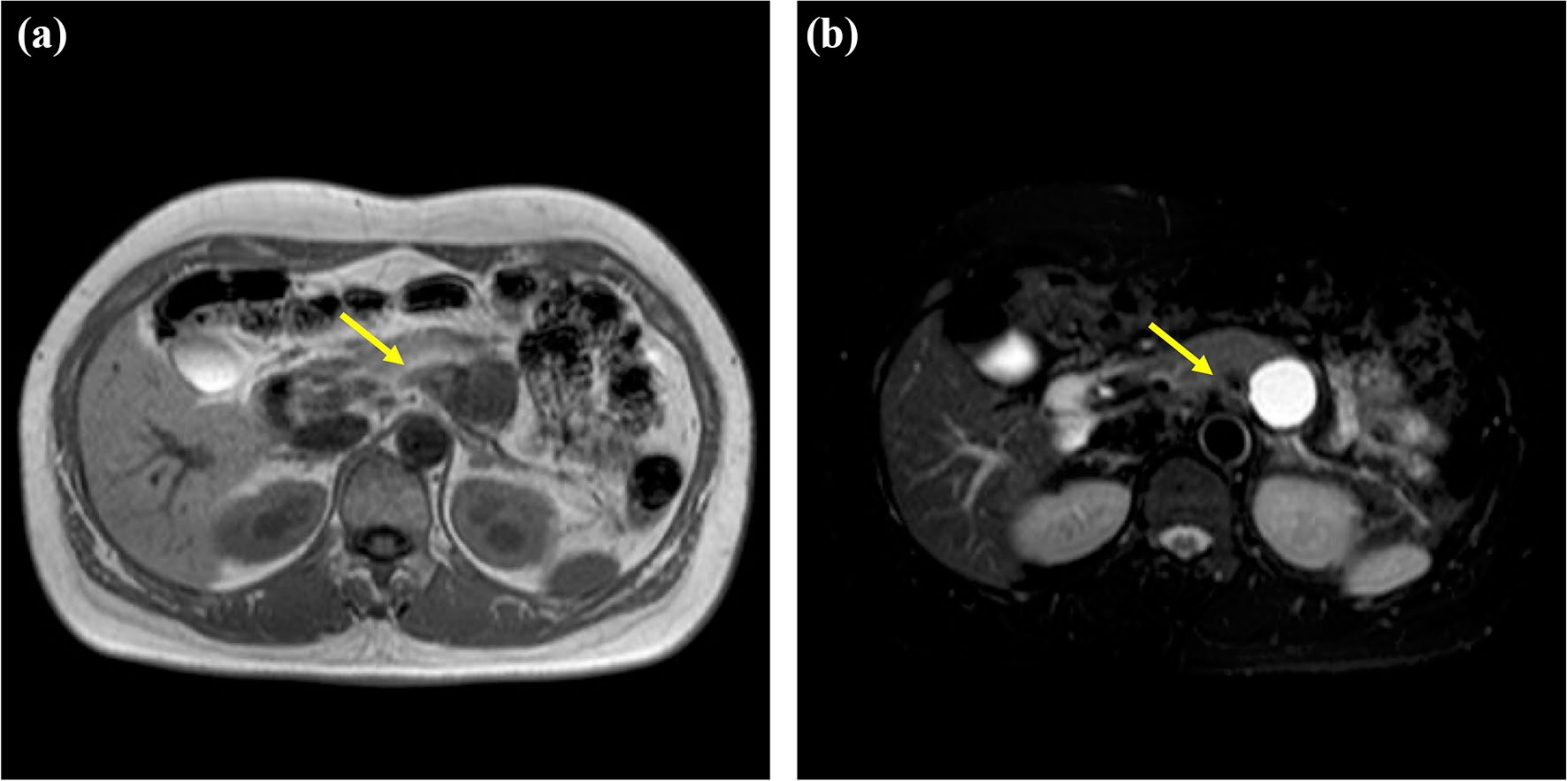
Magnetic resonance imaging showed a 3 cm-diameter unilocular cystic lesion in the pancreatic body, and a soft tissue lesion around the splenic artery (yellow arrows) that was hypointense on T1-weighted (**a**) and hypointense on T2-weighted images (**b**)

**Fig. 4:**
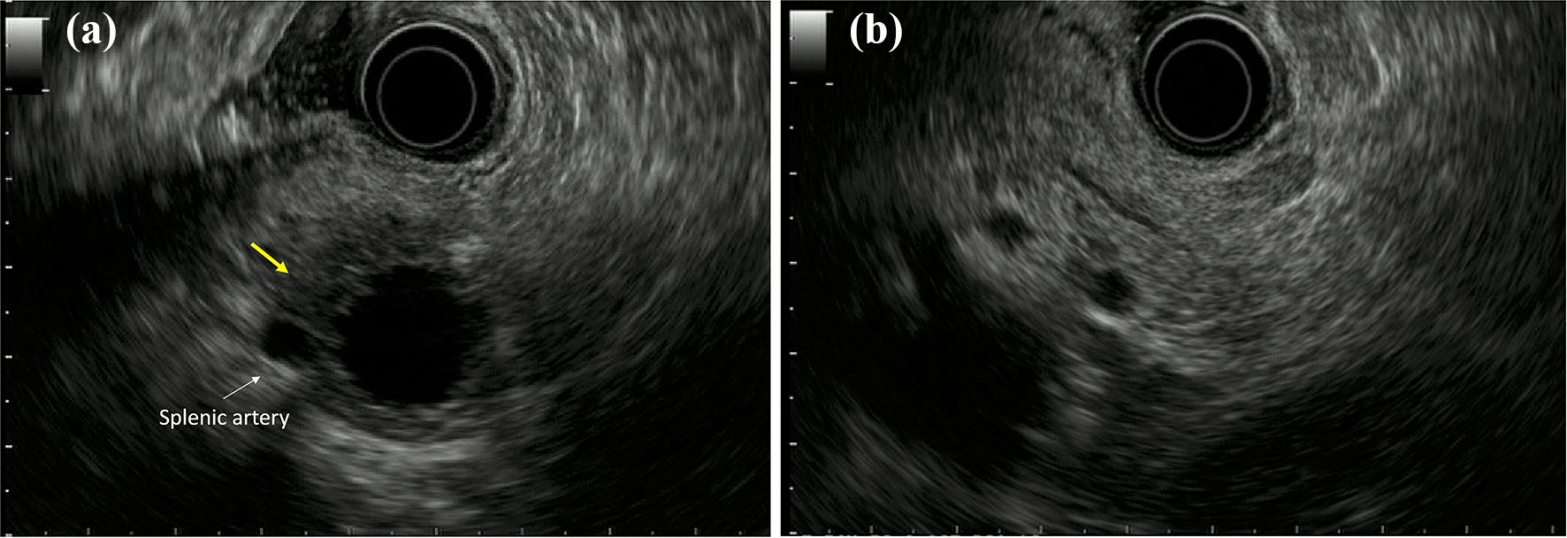
**a** Endoscopic ultrasound showed partial thickening of the cyst wall and a low-echoic area with indistinct boundaries between the splenic artery and the cyst (yellow arrow). **b** There were early chronic pancreatitis findings (foci and strand) in the pancreatic parenchyma around the cyst

**Fig. 5:**
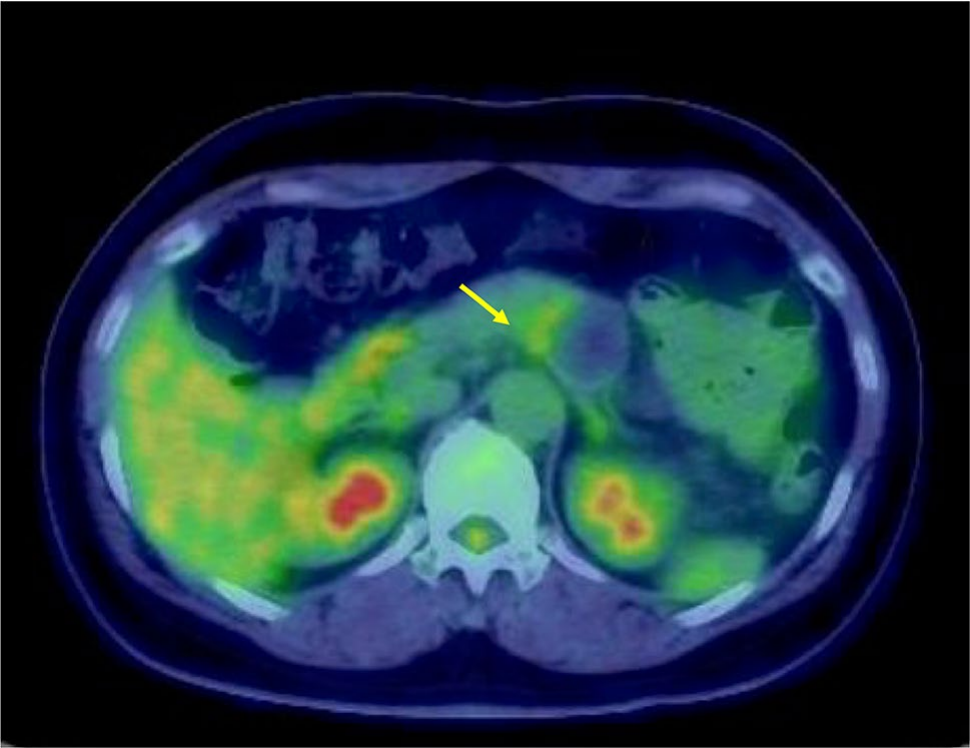
^18^F-fluorodeoxyglucose (FDG)-positron emission tomography combined with a computed tomography scan showed mild ^18^F-FDG accumulation in the papillary side of the cystic lesion (maximum standardized uptake value, 3.5) (yellow arrow)

Enhanced CT revealed a 3-cm large unilocular cystic lesion in the pancreatic body and a soft tissue lesion around the splenic artery on the papillary side, suggesting encasement (Fig. 2). Magnetic resonance imaging (MRI) showed a 3-cm diameter unilocular cystic lesion in the pancreatic body, and the soft tissue lesion around the splenic artery was hypointense on T1-weighted and hypointense on T2-weighted images (Fig. 3a, b). There was no dilation of main pancreatic duct. Endoscopic ultrasound (EUS) showed partial thickening of the cyst wall and a low-echoic area with indistinct boundaries between the splenic artery and the cyst (Fig. 4a). There were early chronic pancreatitis findings (foci and strand) in the pancreatic parenchyma around the cyst (Fig. 4b). ^18^F-fluorodeoxyglucose (FDG)-positron emission tomography combined with CT scan showed mild ^18^F-FDG accumulation in the papillary side of the cystic lesion (maximum standardized uptake value, 3.5) (Fig. 5).

**Fig. 6:**
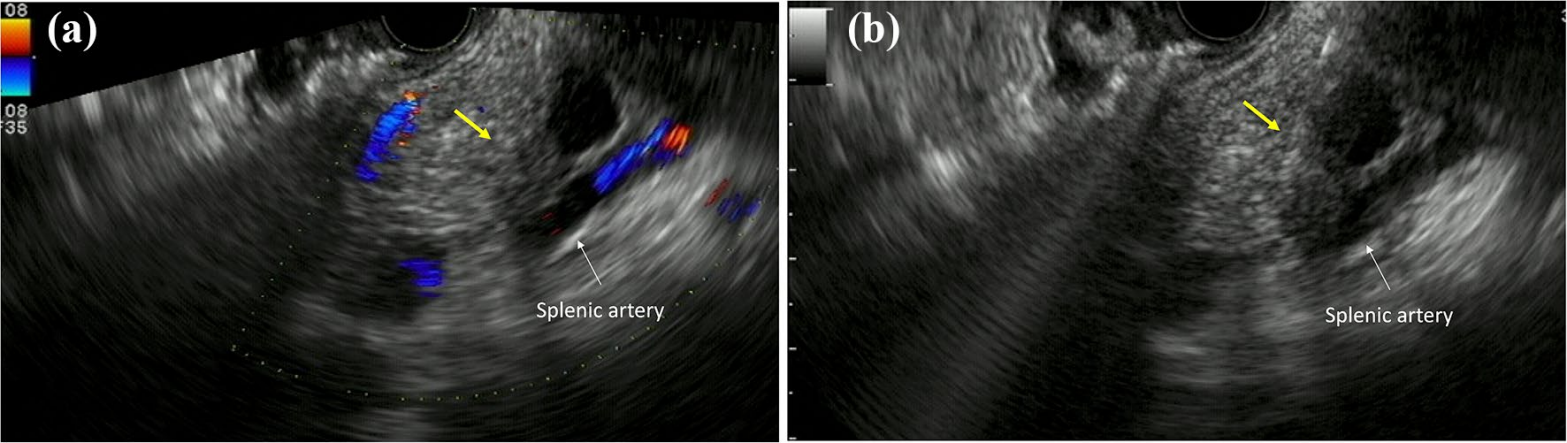
EUS-guided fine-needle aspiration (FNA) was performed for the soft tissue lesion on the papillary side of the cystic lesion around the splenic artery (yellow arrows) (**a**, **b**)

EUS-guided fine-needle aspiration (FNA) was performed for the soft tissue lesion on the papillary side of the cystic lesion (Fig. 6a, b), considering the possibility of invasive pancreatic cancer with retention cyst or invasive carcinoma arising in MCN. However, both pathological and cytological findings were negative for malignancy. The patient denied the operation under the negative for malignancy, although invasive pancreatic cancer was still possible. We, therefore, opted for short-term clinical follow-up.

**Fig. 7:**
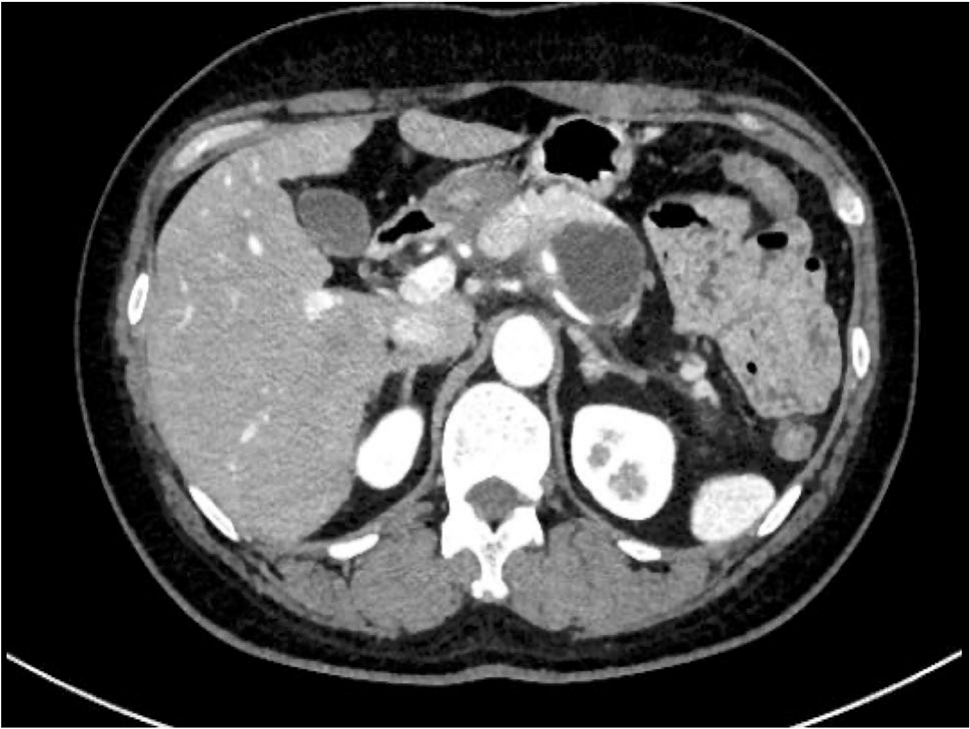
Computed tomography 3 months later showed a slight increase in cyst diameter

**Fig. 8:**
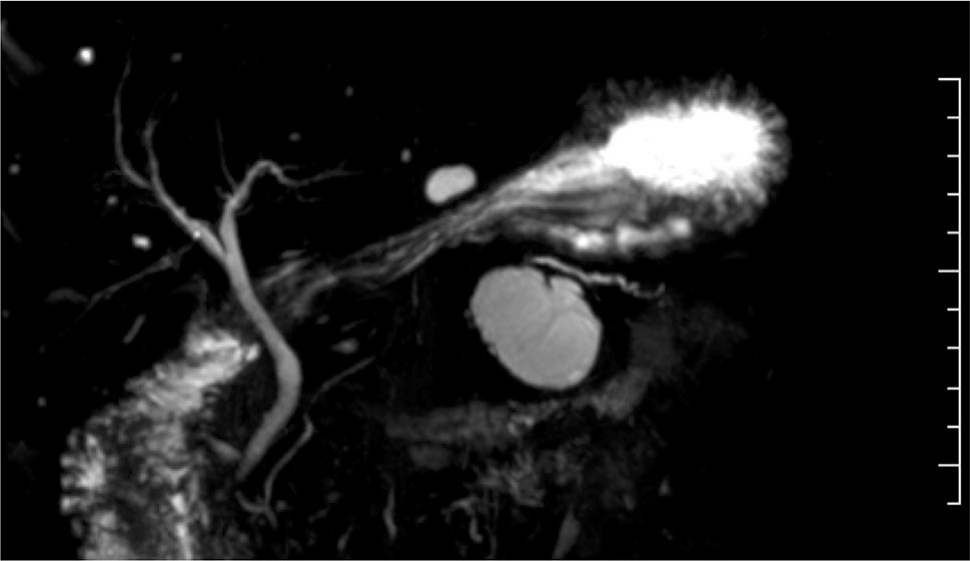
Magnetic resonance cholangiopancreatography (MRCP) showed the appearance of a septum-like structure in the cyst. MRCP also revealed that the main pancreatic duct of the caudal side of the cyst slightly dilated (Fig. 8)

**Fig. 9:**
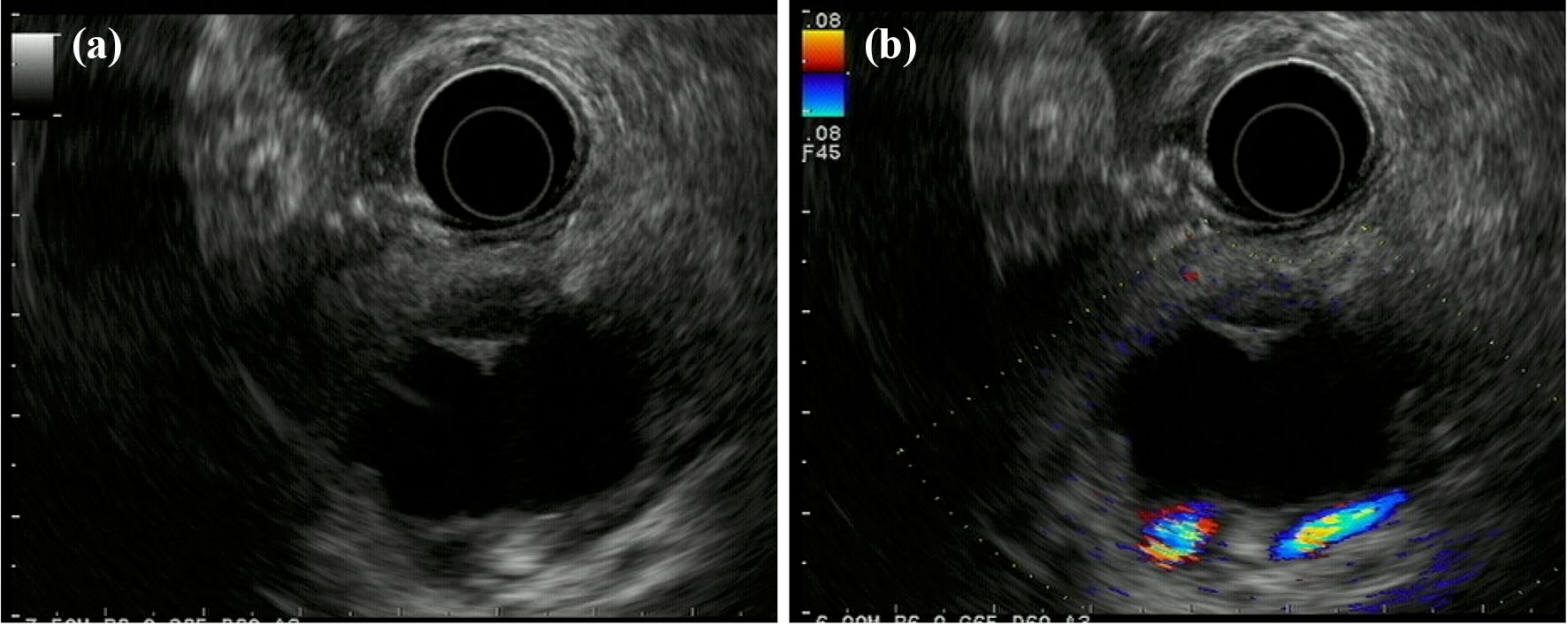
Endoscopic ultrasound showed a cyst-in-cyst structure (**a**, **b**)

CT scan 3 months later showed a slight increase in cyst diameter (Fig. 7), and magnetic resonance cholangiopancreatography (MRCP) showed the appearance of a septum-like structure (Fig. 8). MRCP also revealed that the main pancreatic duct of the tail side of the cyst slightly dilated (Fig. 8). EUS showed a cyst-in-cyst structure (Fig. 9a, b). We suspected MCN with malignant component, and the patient underwent a distal pancreatectomy.

**Fig. 10:**
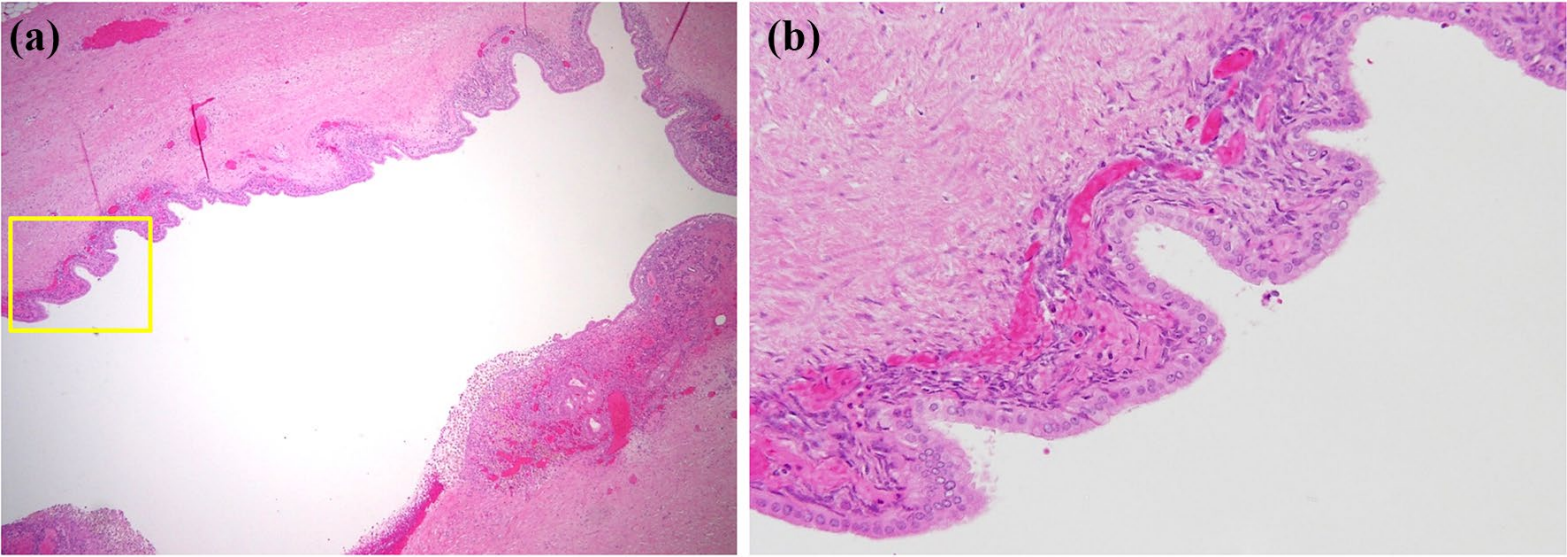
Histologically, the cyst was fibrous (**a**) and the internal surface was covered with low-grade atypical columnar epithelium (**b**)

**Fig. 11:**
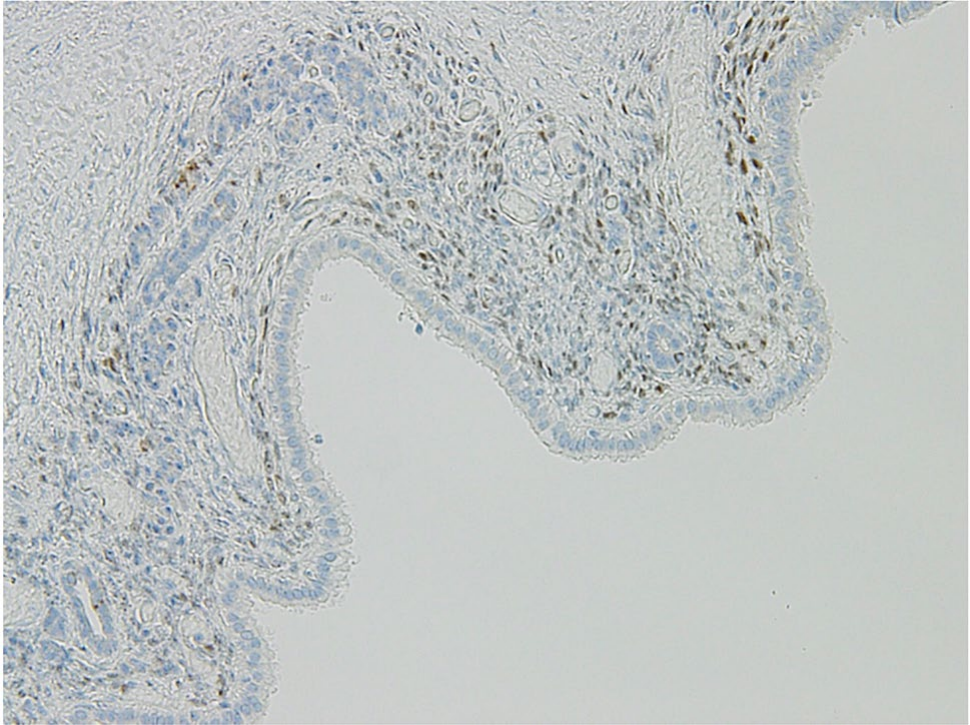
Estrogen receptor-positive spindle-shaped cells were observed under the columnar epithelium

**Fig. 12:**
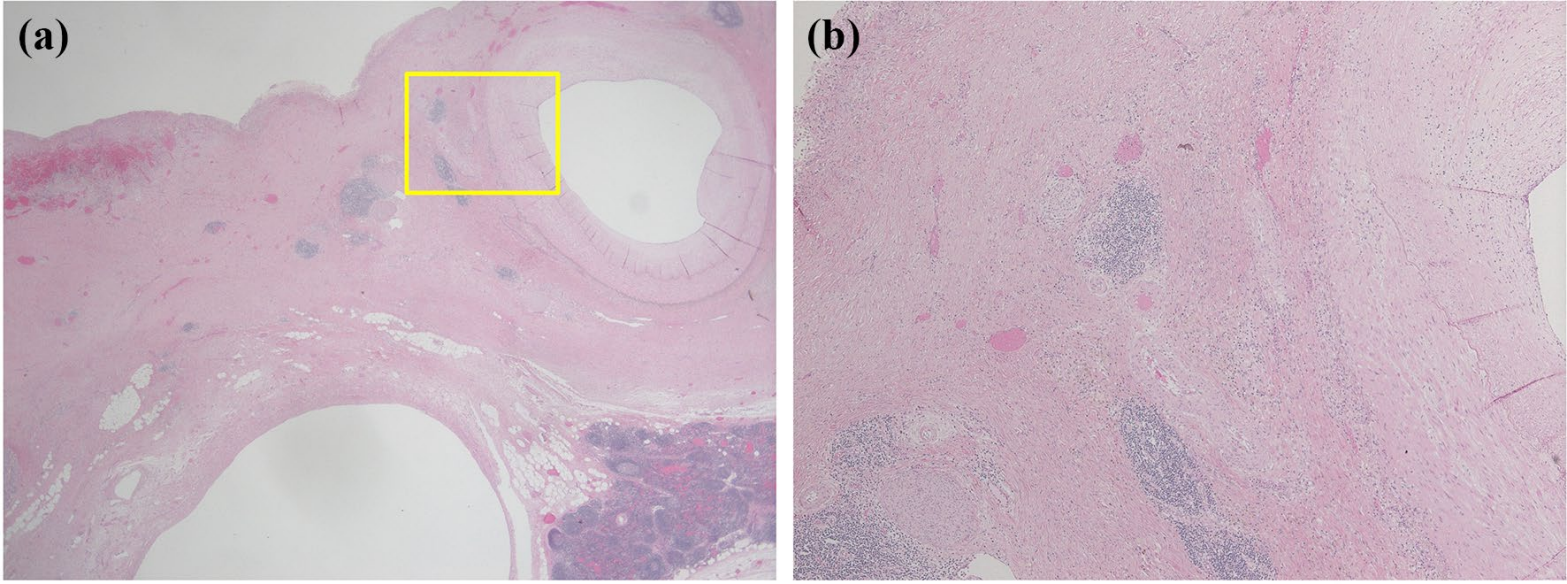
Soft tissue shadows around the splenic artery were considered to indicate fibrosis (**a**) and infiltration of inflammatory cells (**b**)

Histologically, the cyst was fibrous and the internal surface was covered with low-grade atypical columnar epithelium (Fig. 10a, b). Estrogen receptor-positive spindle-shaped cells were observed under the columnar epithelium (Fig. 11). The cells were considered to be ovarian-like stroma (OS), and the patient was diagnosed with low-grade atypical MCA. Soft tissue shadows around the splenic artery were considered to indicate fibrosis and infiltration of inflammatory cells (Fig. 12a, b). After distal pancreatectomy, the patient’s back pain was completely resolved and her postoperative course was uneventful.

## Discussion

MCNs usually involve unilocular or multilocular cystic lesions, often in the form of a cyst-in-cyst. The differential diagnosis of cystic lesions includes cystic tumors such as intrapapillary mucinous neoplasm and serous cystic neoplasm, as well as non-neoplastic true cysts, pseudocysts, and cystic degeneration of solid tumors [1]. The typical findings of MCN include a multilocular cyst with a cyst-in-cyst appearance, and a diagnosis of MCN is relatively straightforward in such cases. A ‘cyst-in-cyst’ appearance involves a cyst with internal septation, in contrast to a ‘cyst-by-cyst’ appearance, which is a characteristic feature of intraductal mucinous papillary neoplasms [4–6]. However, MCNs do not necessarily present with typical findings. In the current case, the initial examinations revealed a unilocular cyst in the pancreatic body, but no septum or nodule was detected inside. Moreover, a soft tissue shadow was seen around the splenic artery on the papillary side of the main lesion, which resembled invasive pancreatic cancer with retention cyst or pseudocyst. Because both the pathological and cytological findings from the EUS-FNA were negative for malignancy, we decided to carry out short-term clinical follow-up, and morphological changes, including a cyst-in-cyst appearance, became evident within 3 months. The patient was, therefore, diagnosed with MCN.

The main complaint in the present patient was epigastric and back pain, which can be caused by localized inflammation around the splenic artery. In this case, no bleeding was observed, and it was not clear if there was a rapid increase in cyst diameter, either of which can cause pain. Although most MCNs are asymptomatic [3], Yamao et al. reported that 6.5% of MCNs presented with acute pancreatitis, which can cause the symptoms [4]. Shioyama et al. reported a case of MCN with short-term morphological changes associated with recurrent pancreatitis [7]. In their case, the main pancreatic duct on the caudal side of the MCN was dilated, and MCN might compress the pancreatic duct, resulting in pancreatitis. The current patient had slight dilation of the pancreatic duct on the caudal side of the lesion, and it was, therefore, likely that her pain was caused by compression of the pancreatic duct. Schofield et al. reported that MCN developed as a result of chronic pancreatic inflammation associated with expression of hypoxia-inducible factor (HIF2α) and additional mutations in the *KRAS* gene [8]. Tissue hypoxia controls cell differentiation in the embryonic pancreas, and HIF2α expression is induced in chronic pancreatitis. If HIF2α is involved, it is possible that the inflammatory changes around the splenic artery observed in this case were caused not only by compression due to the increased size of the MCN, but also by factors such as HIF2α. No similar cases have been reported, and the mechanism of inflammation in this case is thus unclear. However, it is hypothesized that local pancreatitis might have occurred around the splenic artery as a result of the expression of HIF2α in MCN.

MCNs have malignant potential, and surgical resection is usually recommended [9] and can lead to a cure [10]. According to the pathological classification in the seventh edition of the General Rules for the Study of Pancreatic Cancer published in 2016, the Japan Pancreatic Society classified MCN into cystadenoma, noninvasive cystadenocarcinoma, and invasive adenocarcinoma [11]. Park et al. found that mural nodules on CT scans and elevated serum CA19-9 concentrations were significant predictors of malignancy [12]. Ohtsuka et al. reported on a long-term retrospective nationwide study of MCNs with OS and identified five factors predicting malignancy of MCNs: age 56 years or older, high serum CEA level, high CA19-9 level, tumor size ≥ 51 mm, and the presence of mural nodules [13]. However, Sawai et al. reported a rare case of a moderately differentiated invasive ductal carcinoma measuring up to 0.5 cm in diameter in the superficial layer of the cyst wall in the OS [14]. Lewis et al. found that invasive carcinoma confined to the OS of MCNs had an excellent prognosis [9]. It is important to resect MCNs thoroughly, and the recurrence in one patient in their series with incompletely resected MCN suggests that more extensive invasion might have remained in the unsampled tissue. However, most patients with minimally invasive adenocarcinoma arising in an MCN were cured, particularly if the neoplasms were completely resected histologically. In the current case, we suspected MCN with malignant component throughout the preoperative examination, and accordingly performed a distal pancreatectomy.

## Conclusion

We experienced an unusual case of resected MCA with atypical features of unilocular cyst and soft tissue shadow around the splenic artery. The soft tissue shadow around the splenic artery was difficult to distinguish from an invasive pancreatic ductal carcinoma with retention cyst or an invasive component of malignant MCN, and was difficult to diagnose as benign, even though the EUS-FNA pathology/cytology findings were negative. We hypothesize that the MCN might have caused local pancreatitis and inflammation around the splenic artery in our case.

## References

[1] Suzuki A, Atomi Y, Sugiyama M (2004). Cystic neoplasm of pancreas; a Japanese multiinstiutional study of intraductal papillary mucinous tumor and mucinous cystic tumor. Pancreas.

[2] Klöppel G, Solcia E, Longnecker DS, Capella C, Sobin L. Histological typing of tumors of the exocrine pancreas. WHO, Springer-Verlag, Berlin, 1996.

[3] Naveeda S, Qaria H, Bandayb T (2014). Mucinous cystic neoplasms of pancreas. Gastroenterol Res.

[4] Yamao K, Yanagisawa A, Takahashi K (2011). Clinicopathological features and prognosis of mucinous cystic neoplasm with ovarian-type stroma: a multi-institutional study of the Japan pancreas society. Pancreas.

[5] Tanaka M, Chari S, Adsay V (2006). International consensus guidelines for management of intraductal papillary mucinous neoplasms and mucinous cystic neoplasms of the pancreas. Pancreatology.

[6] Ishikawa T, Haruta J, Yamaguchi T (2015). A case of mucinous cystic neoplasm of the pancreas misdiagnosed as a pancreatic pseudocyst at the initial exam and resected after a 2-year follow-up. J Med Ultrason.

[7] Shioyama E, Mitoro A, Ogawa H (2019). A pancreatic mucinous cystic neoplasm undergoing intriguing morphological changes over time and associated with recurrent pancreatitis: a case report. Medicine.

[8] Schofield HK, Tandon M, Park MJ (2018). Pancreatic HIF2α stabilization leads to chronic pancreatitis and predisposes to mucinous cystic neoplasm. Cell Mol Gastroenterol Hepatol.

[9] Lewis GH, Wang H, Bellizzi AM (2013). Prognosis of minimally invasive carcinoma arising in mucinous cystic neoplasms of the pancreas. Am J Surg Pathol.

[10] Scott J, Martin I, Redhead D (2000). Mucinous cystic neoplasms of the pancreas: imaging features and diagnostic difficulties. Clin Radiol.

[11] Japan Pancreatic Society: classification of pancreatic carcinoma, 7th edn. Kanehara & Co., Ltd.: Tokyo, Japan, 2016.

[12] Park JW, Jang JY, Kang MJ (2014). Mucinous cystic neoplasm of the pancreas: is surgical resection recommended for all surgically fit patients?. Pancreatology.

[13] Ohtsuka T, Nakamura M, Hijioka S (2020). Prediction of the probability of malignancy in mucinous cystic neoplasm of the pancreas with ovarian-type stroma: a nationwide multicenter study in Japan. Pancreas.

[14] Sawai H, Kurimoto M, Koide S (2019). Invasive ductal carcinoma arising in mucinous cystic neoplasm of pancreas: a case report. Am J Case Rep.

